# Ceramide in Type 2 Diabetes and Obesity: Modulation by Nutrients and Dietary Patterns and Opportunities to Prevent and/or Manage Metabolic-Related Conditions

**DOI:** 10.3390/metabo16040265

**Published:** 2026-04-14

**Authors:** Melania Gaggini, Adrian Florentin Suman, Cristina Vassalle

**Affiliations:** 1 Institute of Clinical Physiology, National Research Council, 56124 Pisa, Italy; adrianflorentinsuman@cnr.it; 2 Fondazione G Monasterio, 56124 Pisa, Italy

**Keywords:** ceramides, type 2 diabetes, obesity, nutrition, dietary intervention

## Abstract

Ceramides, sphingolipids produced from fatty acids linked to sphingosine and an amide, are structural elements of cellular membranes and lipoproteins. These molecules also retain biological effects in key cellular pathways such as oxidative stress and inflammation, apoptosis, and fibrosis, with a role in the onset and development of many pathophysiological conditions, including obesity, diabetes, and insulin resistance. Increasing evidence suggests that different nutrients and dietary patterns may affect ceramide levels, both negatively (e.g., fructose and the Western diet), whereas others improve the ceramide profile (e.g., ω-3 PUFAs, resveratrol, vitamin D, and the Mediterranean and the Nordic diets). Thus, ceramide nutritional modulation could represent a simple, additive, and reliable tool to improve metabolic health. This review focused on the role of ceramides in the pathophysiology of diabetes and obesity, as well as their pathogenetic mechanisms of action. Ceramides are increasingly recognized as “dynamic metabolic interfaces” linking nutrition and disease. This review aims to address a critical gap by synthesizing recent evidence on how dietary interventions, in addition to pharmacological approaches, can specifically target the enzymatic pathways involved in ceramide synthesis to enhance metabolic health. Thus, this review offers a concentrated analysis of the response of specific ceramide species, such as Cer16:0 and Cer18:0, to distinct dietary factors. Additionally, it incorporates emerging evidence on the role of gut microbiota in the biotransformation of sphingolipids, thereby adding a contemporary dimension to the established nutritional perspective.

## 1. Introduction

**Figure 1:**
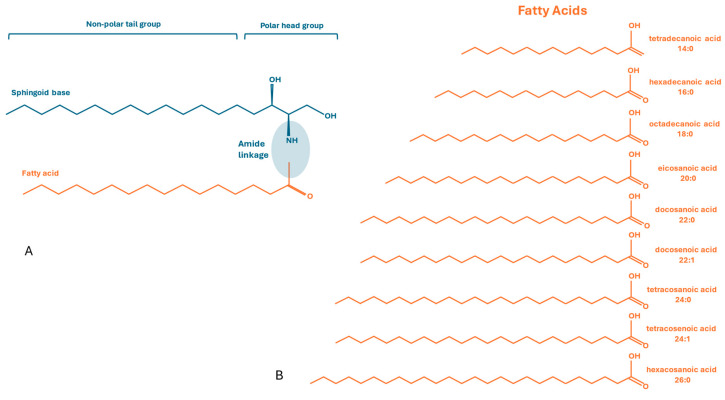
(**A**): General structure of Cers, a sphingoid base formed by a non-polar tail group, and a polar head group amide-bonded to a fatty acid. (**B**): Most common fatty acids represented in Cer lipid profile.

Ceramides (Cers) are one of the main represented classes of sphingolipids, composed of a sphingoid base, a long-chain alcohol that is amide-bonded to a fatty acid with variable length (C2–C34). Cer has two main parts: sphingoid base (often fixed), most commonly d18:1 (18 carbons, 1 double bond; sphingosine), and a fatty acid (acyl chain) that varies in length and saturation (e.g., 16:0, 18:0, 24:1) (Figure 1) [1].

Cers are produced via three main synthesis pathways: the catabolic pathway (sphingomyelin hydrolysis) and the de novo and the salvage pathways (Table 1). Cers are produced in the endoplasmic reticulum (ER) through the action of six ceramide synthases (CerS1–6), each one with specific fatty acyl-CoA preferences, consequently producing ceramides with different chain lengths. The modulation of the enzymes involved in ceramide synthesis aroused interest because it makes it possible to induce or decrease the production of specific ceramide species. A cytosolic lipid transport protein (CERT) delivers ceramides from the ER to the Golgi apparatus, where they act as precursors for synthesizing more complex sphingolipids and glycosphingolipids.

Cers have a structural role in cell membranes and lipoproteins together with biological effects involved in signaling patterns controlling several key cellular pathways, including insulin signaling and resistance, inflammation, oxidative stress, and apoptosis. In particular, Cers, being part of the membranes, take part in lipid rafts, affecting the distribution of receptors on the surface, as well as the stability of the cell membrane itself [2]. Depending on the acyl chain length, ceramides produce different pathophysiological effects and accumulate differentially within each cell type and cell compartment, which may cause adverse consequences associated with the onset and development of metabolic diseases [3]. Accordingly, the malfunction of Cer metabolism has been found to be closely associated with many pathophysiological conditions such as obesity, insulin resistance (IR), and type 2 diabetes (T2D). Also, the ratio between specific ceramides has shown value in the relationship with cardio metabolic risk and prognosis. In this context, CERT1 is a Cer score that stratifies patients based on the quantification of Cer(d18:1/16:0), Cer(d18:1/18:0), Cer(d18:1/24:1), and their ratio to Cer(18:1/24:0). The stratification in four groups is proportional to the risk of cardiovascular disease (CVD). Another score is the CERT2 that considers not only ceramides but also phosphatidylcholine (PC), in particular PC 16:0/16:0 and PC 16:0/22:5, and can predict inflammation, dyslipidemia, and cardiovascular death [2]. Research indicates that specific ceramide profiles are linked to adverse outcomes, as significant biomarkers of metabolic dysfunction [4]. In particular, long-chain Cers (e.g., Cer d18:1/16:0, Cer d18:1/18:0, and Cer 18:1/24:1) and lipid profiling scores (e.g., CERT1, CERT2, and Cer/sphingomyeline) are closely associated with IR, T2D, and adverse cardiometabolic outcomes [5].

The levels of blood circulating Cers are modified by nutritional factors and dietary patterns (e.g., the adoption of the Nordic or the Mediterranean diets may decrease Cer24:0 and Cer18:0 levels, while the Cer24/Cer16 increases), while processed diets containing high saturated fats and sugars result in an unfavorable increase in specific Cer species (e.g., Cer18:0, Cer20:0, Cer22:0, Cer24:0) with a decrease in others (e.g., Cer26/Cer16) [6,7]. Thus, the nutritional modulation of enzymes involved in Cer metabolism might represent an effective and straightforward approach for the prevention and management of metabolic disorders, as Cers act as a dynamic metabolic interface between nutrition and disease. This highlights the strong rationale for further investigating these mechanisms to advance future strategies aimed at maintaining metabolic health and improving public health management [8,9].

Accordingly, this review aims to discuss available evidence on the impact of Cers in T2D and obesity, trying to address a critical gap by synthesizing recent evidence on how dietary interventions, in addition to pharmacological approaches, can specifically target the enzymatic pathways involved in ceramide synthesis, on the opportunity of translation to the clinical setting, and on the potential contribution to develop precision-based nutritional interventions to reduce ceramide levels and mitigate metabolic-related diseases.

## 2. Role of Ceramides in the Onset and Progression of T2D

The pathological mechanism related to T2D development is due to IR, β-cell dysfunction, the activation pro-inflammatory and oxidative stress pathways, and other contributory factors, such as genetic features. Accordingly, the ceramide concentration is associated with the alteration of glucose metabolism and consequently with IR [10].

In an observational cross-sectional study, the role of circulating ceramide in body adiposity and IR in T2D patients and healthy subjects was investigated. In a total of 84 subjects with T2D and 75 nondiabetics (controls), the concentration of ceramide was found to be significantly correlated with the Homeostatic Model Assessment for Insulin Resistance (HOMA)-IR (r = 0.24), β cell function (r = −0.34), and the risk of CVD (r = 0.24) in subjects with T2D; moreover, fasting plasma glucose (OR = 1.83, *p* = 0.01) and ceramide concentrations (OR = 1.05, *p* = 0.01) were significant predictors of IR between T2D patients [11]. In the Mayo Clinic Study of Aging cohort, 1423 adults (47% women; median age 72 years) were included, with 222 prevalent and 37 incident cases of T2D. The associations between ceramides and both prevalent T2D at study enrollment and incident T2D during a 6.2-year follow-up were investigated. In particular, higher levels of Cer16:0 were associated with lower odds of prevalent T2D (odds ratio-OR 0.84 [confidence intervals, CI 0.71–0.99]; *p* = 0.03), whereas Cer18:0 and the ratio Cer18:0/16:0 and Cer18:0/24:0 were associated with higher odds, (OR 1.27 [1.06–1.42]; *p* = 0.01); (OR 1.41 [1.22–1.62]; *p* < 0.001) and (OR 1.22 [1.05–1.41]; *p* = 0.01). The ratio Cer18:0/16:0 and Cer18:0 was associated with an increased risk of incident T2D in Cox hazard regression models, (hazard ratio, HR 1.63 [1.26–2.10]; *p* < 0.001) and (HR 1.53 [1.12–2.08]; *p* = 0.01) [12].

In another study (2072 middle-aged and older American subjects included in the MIDUS cohort), the relationship among ceramide levels, insulin resistance, and T2D prevalence was evaluated. The results showed that Cer18:0 and Cer22:0 were associated with IR and a higher prevalence of T2D, whereas three lactosylceramides (LCER 14:0, 16:0, and 24:1) were inverse correlated with both IR and T2D; from these data, two sphingolipid scores were developed, which consistently predicted a reduced risk of T2D incidence in the PREDIMED cohort in a prospective analysis (250 cases and a random sample of 692 participants, with 3.8 years of median follow-up) [13].

Plasma ceramides (Cer16:0, Cer18:0, Cer24:0, Cer24:1) and the CERT1 score were measured in a prospective study that included 1903 outpatients with T2D patients with the purpose of evaluating whether ceramides predict the risk of mortality in T2D patients. Results showed that subjects with higher CERT1 scores (≥7) had a 1.86-fold (95% CI, 1.30–3.65) increased higher risk of all-cause death compared to those with a low score (≤2), after an adjustment for cardiorenal risk factors. Specifically, patients with a CERT1 score in the high-risk category had significantly higher risks of cardiovascular, as well as non-cardiovascular, death (after adjustment for demographic and cardiometabolic risk factors) when compared to patients in the low-risk category. Moreover CERT1 improved the prediction of the 10-year risk of all-cause death [14].

Untargeted and targeted mass spectrometry was used for analyzing the blood lipid profiles of Chinese subjects with newly diagnosed T2D or without impaired fasting glucose/impaired glucose tolerance (IFG/IGT; preT2D), or overt T2D. The results show that Cer(24:0) has higher predictive power in preT2D and T2D compared with Cer(23:0) and Cer(22:0) [15].

In a study conducted on 435 American-Indian (Strong Heart Study and Strong Heart Family Study) and in 1902 participants from the Strong Heart Family Study, the associations of 15 blood ceramides and sphingomyelin (SM) species with incident T2D were evaluated. In cases of incident T2D across the studies (*n* = 446), the increase in T2D risk was associated with a higher circulating concentration of ceramides containing stearic acid (Cer18:0), arachidic acid (Cer20:0), and behenic acid (Cer22:0) [16].

Currently, procedures for ceramide measurement are not yet widespread and accessible to all laboratories, and some practical difficulties must still be overcome (e.g., costs, standardization of the procedures, complexity of instruments, and skilled professionals), together with the still poor understanding of the intricate network of relationships involved in ceramide metabolism [17]. However, the possibility to modulate ceramide is highly relevant to both clinical and translational medicine, representing a key research target for preventing and counteracting T2D-related metabolic issues.

## 3. Association Between Ceramide Profiles and Obesity

Different species of ceramides can accumulate within distinct cells and cellular compartments, which can then determine distinct pathophysiological effects, causing a number of adverse events associated with obesity (e.g., lipotoxicity, inflammation, mitochondrial activity, and IR) [3]. In subjects considered as having metabolically healthy obesity (MHO) and metabolically unhealthy obesity (MUO), the potential biomarkers able to differentiate these subtypes of obesity were investigated. In particular, a lipidomic evaluation of ceramide in the serum showed that in the MHO group, Cer24:1 levels were lower than in the MUO group. As Cer24:1 increased, a significant decrease in insulin sensitivity was observed, accompanied by a worsening of metabolic parameters; serum Cer24:1 values were independently correlated with MUO. Moreover, some species of ceramides (Cer 18:0/18:0 and Cer 18:1/16:0) decreased in obese subjects [4].

In an interesting study on the relationships between ceramide species concentrations in the liver, plasma and very low-density lipoprotein (VLDL) particles of subjects with obesity (*n* = 25) were evaluated. Results obtained showed a positive correlation between the proportion of Cer14:0, Cer18:0, Cer20:0, and Cer24:1 ceramide in the liver and whole plasma (γ = 0.491, *p* = 0.013; γ = 0.573, *p* = 0.003; γ = 0.479, *p* = 0.015; γ = 0.716, *p* = 0.00006; respectively). Thus, the proportions of whole plasma ceramide subspecies (C14:0, C18:0, C20:0, and C24:1) reflect those of hepatic ceramides in subjects with obesity, indicating their potential as biomarkers of the hepatic ceramide composition [18].

Cer levels, as well as the mRNA expression level for different enzymes related to ceramide production, were analyzed in subcutaneous adipose tissue samples collected from lean non-diabetic, obese-non-diabetic, and obese-diabetic subjects. The authors demonstrated that several species of ceramides were significantly higher in the obese group compared to the normal weight group (C16-dihydro-ceramide, C18-dihydro-ceramide, and Cer24). In addition, stratification of the obese group revealed a significant elevation in the C16-ceramide values. Moreover, serine palmitoyl transferase (SPT1) exhibited a significant fold change in the expression level (obese-T2D group vs. obese-non-T2D subjects), whereas no difference was observed in the expression levels of the other enzymes studied [19]. In 20 nondiabetic obese women with hepatic steatosis, surgical subcutaneous adipose tissue biopsies were studied revealing that Cer24:1 showed increased levels in the inflamed subcutaneous white adipose tissue (WAT) [20]. Moreover, in a study involving obese women, total ceramide levels were found to be higher in visceral tissue compared to subcutaneous WAT, with Cer16:0 and Cer18:0 specifically associated with systemic metabolic abnormalities [21]. Moreover, in a study involving subjects across different body mass indexes (BMI), an increase in most ceramides was recorded in both the subcutaneous and visceral epicardial WAT depot in obese individuals, with a close association between Cer16:0 ceramides in subcutaneous WAT and high HOMA-IR (r = 0.79, *p* < 0.001) [22].

Another study examined the lipid composition of adiposomes in 122 subjects (75 individuals with obesity, 47 lean individuals) in relation to cardiometabolic risk. Subjects with obesity showed elevated adiposome release and shifts in the lipid composition; total Cer levels were significantly higher in subjects with obesity, whereas Cer in lean individuals often contained more carbons and unsaturated bonds [23]. Lange et al. performed a lipidomic analysis in both lean and obese individuals (*n* = 5; BMI = 23.1 ± 1.5 kg/m^2^; age = 68 ± 10.9 years; male/female = 3/2; *n* = 81; BMI = 45.1 ± 1.2 kg/m^2^; age = 45 ± 2.2 years; male/female = 26/55, respectively), in subcutaneous and visceral adipose tissue, confirming that Cer16:0 is the most abundant species in human adipose tissue [24].

To date, data clearly demonstrated that ceramide metabolism is adversely modified in subjects with obesity, as well as that the increase in specific ceramides can have harmful effects in a tissue-specific manner (Table 2). Thus targeting specific CerS enzymes in subjects with obesity (e.g., CerS1 and CerS6) could be an important objective, as it may face obesity-related metabolic disturbance, overcoming possible adverse events related to the global inhibition of ceramide production [25].

**Table 2:** Trend in specific ceramides according to the presence of obesity or T2D.

Study Population	Size of the Study	Endpoint	Ceramide Species	Trend	Ref
Mayo Clinic Study of Aging—elderly subjects	1423	Incidence of T2D	16:0	↓	[12]
Obese cohort compared with lean controls (lean non-diabetic *n* = 20; obese non-diabetic *n* = 66; obese diabetic *n* = 32)	118	Obesity	16:0	↑	[19]
Women with class III obesity (visceral adipose tissue study)	28	Inflammation of adipose tissue/metabolic syndrome	16:0	↑	[21]
Mayo Clinic Study of Aging—elderly subjects	1423	Incidence of T2D	18:0	↑	[12]
MIDUS cohort (middle-aged and older adults)	2072	Association with insulin resistance and higher prevalence of T2D	18:0	↑	[13]
Strong Heart Study (SHS) and Strong Heart Family Study (SHFS)	435 and 1902	Increased risk of T2D	18:0	↑	[16]
Strong Heart Study (SHS) and SHFS	435 and 1902	Increased risk of T2D	20:0	↑	[16]
MIDUS cohort (middle-aged and older adults)	2072	Incidence of T2D	22:0	↑	[13]
SHS and SHFS	435 and 1902	Incidence of T2D	22:0	↑	[16]
Newly diagnosed T2D or normal glucose tolerance cohort	93 (discovery)/440 (validation)	Predictive power for pre-T2D and T2D	22:0	↑	[15]
Newly diagnosed T2D or normal glucose tolerance cohort	93 (discovery)/440 (validation)	Predictive power for pre-T2D and T2D	24:0	↑	[15]
Obese cohort vs. controls (lean non-diabetic *n* = 20, obese non-diabetic *n* = 66, obese diabetic *n* = 32)	118	Obesity	24:0	↑	[19]
Metabolically healthy vs. metabolically unhealthy obesity	77	Metabolically unhealthy obesity	24:1	↑	[4]
Nondiabetic obese women	20	Inflamed subcutaneous white adipose tissue	24:1	↑	[20]
Mayo Clinic Study of Aging—elderly subjects	1423	Incidence of T2D	CERT1 score	↑	[13]

**Up arrow (**↑**): rising trend, down arrow (**↓**): falling trend**

### Mechanism of Action of Ceramides in the Development of T2D and Obesity

In view of available evidence, ceramide levels are closely related to IR and T2D (Table 2). Beyond oxidative stress and inflammation, the likely impairment of insulin signaling and the promotion of pancreatic cell suffering and death represent key events in this relationship [17]. The mechanism of IR is as follows: ceramides negatively regulate the action of insulin through the inhibition of Akt/protein kinase B (PKB), a serine/threonine kinase, which are important for glucose uptake and anabolic metabolism. In 3T3-L1 preadipocytes, ceramide blocks insulin stimulation of Akt/PKB via two independent mechanisms. First, it specifically blocks the translocation of Akt/PKB, but not its upstream activator phosphoinositide-dependent kinase-1, to the plasma membrane, and the second mechanism reveals that ceramide stimulates the dephosphorylation of Akt/PKB by protein phosphatase 2A [26]. The inhibition of Akt’s transport to the plasma membrane disrupts the Akt/PKB pathway, reduces GLUT 4 expression levels, and perturbs the glucose transporter pathway, leading to IR [27]. Also, ceramides activate protein kinase C (PKC), which phosphorylates Thr34 on Akt, leading to an increase in the recruitment of Akt to the cytoplasmic membrane, impairing insulin signaling [28]. The inhibition of AKT also results from the Toll-like receptor 4 (TLR4)-mediated upregulation of genes involved in ceramide synthesis, mediated by the activation of IKKβ and NF-κB, which ultimately leads to increased ceramide levels [29]. Another important factor associated with the development of IR occurs when ceramides induce apoptosis in islet β-cells, which have an important role in the production of insulin. Ceramides increase the release of cytochrome c, causing the apoptotic pathway in islet β cells and are also mediators of fatty acid-induced cytotoxicity in β cells [30]. IR and obesity are closely related since they are associated with intensified lipid accumulation, including sphingolipids, as well as ceramides. In particular, pro-inflammatory TLR4 activation (by saturated fatty acids or lipopolysaccharide) [31] promotes the upregulation of enzymes involved in de novo ceramide biosynthesis, such as serine palmitoyltransferase and ceramide synthase. The activation of TLR4 leads to increased inflammation and ceramide production associated with the development of obesity. A hallmark of obesity is the inflammation of adipose tissue characterized by the increased recruitment and activation of macrophages inside the adipose tissue. This process leads to increased expression and secretion of inflammatory cytokines, including tumor necrosis factor-α (TNF-α), that promote increased ceramide levels [32]. Several studies have shown that the contents of ceramides in adipose tissue are elevated in individuals with IR independent of obesity [20,33]. Instead, in another study, it was found that various ceramide species were increased in the adipose tissues of obese subjects and that the mRNA expression of CERS6 positively correlates with BMI, hyperglycemia, and body fat content, while negatively correlating with the glucose infusion rate during euglycemic-hyperinsulinemic clamps. This type of discrepancy with ceramide levels depends on the different populations, as ceramides can be directly associated with IR and metabolic dysfunction. Not necessarily with increased fat mass, in some populations, they appear to be associated primarily with the obesity phenotype. Ceramides can therefore increase in both T2D and obesity, but through partially different pathophysiological pathways.

## 4. Nutritional and Dietary Issues

Different drugs, commonly used in the cardiometabolic settings (e.g., statins, metformin), are able to affect ceramide biosynthesis, as well as some specific inhibitors targeting ceramide metabolism, revealing interesting potential for therapeutic clinical applications [2]. Specifically, as six fatty acyl selective ceramide synthases are involved in ceramide production, a specific enzymatic modulation can change (increasing/reducing) the production of ceramide species, with specific adverse or protective biological actions; this fact evidences how the enzymatic targeting may represent an innovative therapeutic tool [34]. However, the intricacy of ceramide production and the interactions among various ceramides with beneficial/adverse effects, depending on the synthesis pathways and enzymes involved, makes the development and spread of these drugs challenging [17].

Sphingolipids are contained in different foods, especially dairy products, but also in meats, eggs, fishes, and some vegetables [35]. Moreover, many nutrients, including fats, carbohydrates, polyphenols, vitamins, and proteins, can modify blood sphingolipid and ceramide metabolism. Thus, dietary interventions might represent a simple and reliable strategy to modulate blood ceramide levels useful in different conditions [8], including in subjects with obesity and/or T2D or other cardiometabolic diseases where a healthy eating pattern already represents a key tool for weight loss and glycemic control together with the currently available therapeutic treatments and adequate regular physical activity.

Nutrients: In particular, different clinical results suggest that circulating Cers are modified by saturated and unsaturated fatty acids (SFA and PUFA, respectively; between PUFA especially omega-3 PUFA [e.g., eicosapentaenoic acid and docosahexaenoic acid)]; this evidence is not surprising as fatty acids are fundamental blocks of the ceramide structure. Accordingly, dietary saturated fatty acids, particularly palmitic acid, resulted as the main factors responsible for the increase in ceramides in blood [36]. Thus, increasing the dietary content of palmitate in 18 healthy volunteers induced elevated levels of Cers in the blood (and muscle) both in the fasting and in the fed states; moreover, nearly every serum ceramide species increased in response to the diets enriched in palmitic acid [37]. Moreover, the consumption of a high-saturated-fat meal significantly increased several sphingolipids, including ceramides, together with lactosylceramide, and sphingomyelin-14 in healthy subjects [38].

High saturated fat intake, mainly palmitic acid, induced an increased concentration of ceramide and other lipids (e.g., monohexosylcermides, dihexosylceramides, sphingomyelins, and sphingosine 1-phosphates) in a large Chinese population (2860 subjects; nutrient intake estimated by a validated 159-item food frequency questionnaire); instead, the high intake of polyunsaturated fats, of interest for their anti-inflammatory properties and their beneficial role in obesity-related diseases, was correlated with lower plasma long-chain ceramide levels, as well as protein intake, that were inversely related to sphingolipid levels [39]. Other data suggest that polyunsaturated fatty acids reduced circulating ceramide levels, whereas saturated fatty acid increased blood ceramides in subjects with overweight/obesity [40]. Moreover, overfeeding in healthy subjects (1250 kcal/day for 28 days), beyond clearly leading to weight gain, induced modifications in the blood lipid profile (total ceramide, Cer22:0 and Cer24:0 increased, total diacylglycerol and lysoalkylphosphatidylcholine decreased) [41]. Interestingly, a total of 38 overweight subjects (age 48 ± 2 years, BMI 31 ± 1 kg/m^2^) underwent 1000 extra kcal/day daily excess calories as saturated fat, unsaturated fat, or carbohydrate for 3 weeks. Thus, altogether these results suggest that changes in macronutrients (e.g., reduced saturated fats and increased polyunsaturated fats and proteins) induce modification of the lipidomic profile.

Vitamins (e.g., B6, C, D, and K), polyphenols, and flavonoids, powerful and complementary antioxidant micronutrients, can modulate the metabolism of sphingolipids and ceramide blood levels [42]. Indeed, a mutual crosstalk between vitamin D and sphingolipids has been identified. In fact, it is known vitamin D can influence sphingolipid homeostasis at various levels (e.g., by affecting the hydrolysis of sphingomyelin, S1P receptors and sphingosine kinase 1 and 2 expression; downregulating ceramide kinase, responsible for the synthesis, expression, and content of ceramide-1-phosphate; upregulating ceramides). This evidence has been verified by experimental and clinical data showing the modification of the blood sphingolipid profile by vitamin D administration in T2D, dyslipidemia, and overweight/obese subjects [25]. Conversely, the localization and expression of sphingolipids in lipid rafts may modulate the localization and functioning of vitamin D receptors [34]. In particular, vitamin D supplementation can modulate levels of long-chain ceramides (e.g., increasing ceramide (C18) and dihydroceramide (C18) levels) in T2D subjects [43]. In agreement with these data, vitamin D3 supplementation has been found to increase long-chain ceramide serum levels (C18Cer and C18SM) in African American subjects with overweight/obesity [44]. In an acute myocardial infarction (MI) population, a significant inverse correlation between 25(OH)D and Cer16:0 and Cer18:0 was observed; diabetic/dyslipidemic patients with reduced levels of 25(OH)D (<30 ng/mL) had higher levels of both ceramides with respect to the rest of the population. Moreover, patients with severe hypovitaminosis D (<10 ng/mL) presented the highest levels of the two ceramides. On the other hand, 25(OH)D remained as an independent determinant for Cer16:0 (STD Coeff −0.18, t-Value −2, *p* ≤ 0.05) and Cer18:0 (−0.2, −2.2, *p* < 0.05) after an adjustment for different traditional cardiometabolic risk factors [45].

Resveratrol (RSV) is a polyphenol that has different beneficial actions (e.g., anti-inflammatory, antioxidant, anti-cancer, and neuroprotective effects). Many anti-cancer effects of RSV are ascribed to actions on the sphingolipid and ceramide metabolism; in particular, RSV has been found to increase Cer levels and induce apoptosis in colon, breast, and prostate cancer cells and to downregulate SK1 expression and activity in prostate cancer cells [46]. Moreover, RSV has anti-proliferative effects and favors apoptosis through the inhibition of ceramide catabolism enzymes (acute myeloid leukemia) [47]. In fact, RSV can increase redox reactions, the generation of ceramides, and the expression of apoptosis receptors (e.g., Fas Ligand). Moreover, it also affects mitochondrial pathways inducing apoptosis. Conversely, RSV has an inhibitory effect on different antiapoptotic mediators (e.g., Nuclear Factor κB, NF-κB), cyclooxygenase-2 (COX-2), phosphatidylinositol 3-kinase (PI3K), and mTOR, a key protein kinase [48]. At present, many aspects regarding the balance between the beneficial/adverse effects of RSV on sphingolipids and ceramide metabolism remain largely unknown and unclear. Interestingly, the administration of the polyphenol tyrosol (4-weeks), added in white wine, in subjects at a high risk of CVD, was associated with lower levels of C16:0/C24:0, C18:0/C24:0, and C24:1/C24:0, in parallel to an improvement of endothelial function (evaluated as reactive hyperemia index/augmentation index) [49].

In 169 subjects with dyslipidemia, supplementation with anthocyanin (with antioxidant properties and belonging to the flavonoid group-40, 80, or 320 mg/day for 12 weeks) dose-dependently reduced Cer16:0 and Cer24:0 blood ceramide values and improved the lipid profile [50].

Foods: The intake of some foods has shown associations with changes in the profile and levels of ceramides. Accordingly, the Framingham Offspring Study found a positive association between sugar-sweetened beverage consumption (a primary source of dietary fructose) and concentrations of Cer16:0, Cer22:0, and Cer24:0 in subjects followed over 14 years [51]. Instead, fructose restriction decreased total ceramides and ceramide metabolites (Cer14:0, Cer22:0 and Cer24:0) and improved insulin sensitivity in children with obesity at cardiometabolic risk [52]. Walnut consumption (48 g walnuts for 5 days) improved the lipid profile, including ceramides, hexosylceramides, and sphingomyelins, all proven to be harmful for cardiometabolic health in subjects with obesity [53]. Milk polar lipid-enriched cream cheese consumption (0, 3, or 5 g for 4 weeks) in 58 postmenopausal women decreased serum atherogenic Cer24:1 C16:1 SM and C18:1 SM species [54]. In the D.E.S.I.R. cohort, higher consumption of dairy products (except cheese) was associated with lower total dihydroceramides (direct ceramide precursors) and a trend toward lower Cer in the blood during the follow-up, but only in women (not observed in men) [55]. The consumption of chocolate spread snacks containing different fat types (extra virgin olive oil-EVOO rich in monounsaturated fatty acids or palmitic acid) in healthy young subjects for 2 weeks reduced plasma ceramide Cer16/Cer22:0 and Cer16/Cer24:0 and sphingomyelin C18:0 (*p* = 0.030, *p* = 0.032 and *p* = 0.042, respectively) and preserved insulin levels and insulin sensitivity (HOMA-IR) in the EVOO-, compared to the palm oil-enriched, chocolate spread diet [56].

Dietary patterns: The Mediterranean and Nordic diets (both nutritional patterns characterized by high contents of unsaturated fats), represent whole healthy eating patterns capable of modifying different sphingolipid classes and the cardiometabolic disease risk. In the PREDIMED study (230 incident cases of CVD and 787 randomly selected participants, which were followed for ≤7.4 years), a positive association between baseline blood ceramides and incident CVD was observed; in addition, the Mediterranean diet reduced the cardiovascular disease risk at follow-up in subjects initially showing elevated plasma Cer concentrations [57]. Although the Mediterranean diet may indirectly face the adverse lipid profile through its antioxidant/anti-inflammatory properties (e.g., polyphenol content), fiber intake, and LDL reduction, the lower content of saturated fats (e.g., respect to the Western diet) may also contribute to its beneficial effects. In this context, elevated saturated free fatty acids (FFAs; palmitic acid and stearic acid) stimulate Cer production, which can be instead prevented by polyunsaturated FFA (e.g., arachidonic acid) in an in vitro model [58].

The adoption of a healthy Nordic Diet (high consumption of fish, low-fat milk products, fiber-rich whole grains, vegetables, and fruit) reduced ceramide levels in subjects with obesity Cer22:0, Cer23:0, and Cer24:0 [44]. Moreover, a 4-week vegetarian diet lowered levels of several lipotoxic lipids (including Cer16:0) and improved cardiometabolic risk factors compared with an isocaloric diet (including meat consumption) in patients with coronary artery disease [59].

In patients with recent MI/unstable angina (*n* = 33) and high consumption of fatty fish, rich in omega-3 fatty acids (≥4 times/week for 8-weeks), a decreased blood Cer concentration, together with reduced levels of diacylglycerols and lysophosphatidylcholines, was observed [60]. Dietary intervention for 8 weeks that is focused on increasing the consumption of fruit and vegetables, together with the intake reduction of refined carbohydrates or fat in young adults, beyond beneficial effects on waist circumference, systolic blood pressure, and cholesterol levels, is able to reduce levels of circulating Cer24:0, although an increase in serum Cer16:0 was also observed [61]. This fact reveals the complexity of ceramide interaction and the inability to fully understand the overall biological effects derived by a decrease in a specific ceramide that in turn might increase the amount of other toxic ceramides. In fact, all these nutritional and dietary approaches may represent effective and natural tools to modulate circulating ceramide levels, even in diseased patients, when combined with therapeutic treatment. In this context, Cers may represent a promising biomarker at the crossroad between diet quality and metabolic health, as well as a possible nutritional target to lower the risk of metabolic abnormalities. However, many aspects related to the complexity of ceramide metabolism, the number of interactions between species, and the different regulation of different cellular types still need to be clarified and currently constitute challenges that complicate the exact knowledge of all consequences derived from the implementation of nutritional strategies targeting ceramide metabolism [62]. These factors also contribute to the low reproducibility across populations, also influenced by a number of other variables, which may effectively act as confounding factors (e.g., diet quality, statin use, ethnicity).

Other dietary-related interventions: Interestingly, results are emerging concerning caloric restriction, weight loss (also associated with glucagon-like peptide-1 receptor agonist therapy alone or combined with exercise), and bariatric surgery, which are all able to change the lipidomic status [63,64,65].

Gut microbiota: In a healthy state, the microbial gut is in balance with host cell physiology, regulates the immune status of the host, and contributes to resistance against pathogens and to the maintenance of the intestinal mucosal barrier functionality. The relationships between the gut microbiota and host immunity are intricate, bidirectional, and dependent on the situation; the microbiome drives the development of the host’s innate and adaptive immune system, whereas the host immune system regulates the microbiome composition and contributes to the maintenance of host–microbe symbiosis [66]. Dysbiosis and related immune modulation represent key determinants in the pathogenesis of intestinal disease (e.g., inflammatory bowel diseases, irritable bowel syndrome, and colorectal cancer) [67]. In newborns, different gut microbiota patterns due to early environmental exposures, (e.g., reduced microbial diversity due to cesarean delivery) are associated with different T cell development and immune tolerance, which may predispose the individual to allergic diseases [68,69]. It is known that the gut microbiota directly faces pathogens through multiple actions (e.g., nutrient competition, production of bacteriocins, and contact-dependent inhibition) [70].

In obesity, adverse changes in the gut microbiota increase intestinal permeability and circulating endotoxins, which in turn favor systemic low-grade inflammation and metabolic dysfunction [71]. Conversely, the gut microbiota may metabolize polyunsaturated fatty acids towards metabolites beneficial for the development of obesity and inflammation [72]. In particular, the gut microbiota may represent an additive key determinant in the relationship among the diet, lipid metabolism, and metabolic health. In this context, gut bacteria can produce, as well as bio-transform, sphingolipids, and these capacities affecting lipid metabolism have important effects on host immunological responses and metabolic health. In particular, a number of bacteria may produce sphingolipids (e.g., *Bacteroides* and *Prevotella*, abundant in the human gastrointestinal tract); bacterial sphingolipids, characterized by odd chain lengths (due to presence of branched alkyl chains) have been identified in various tissues (e.g., liver, colon, brain, skin), promoting host symbiosis with effects on host lipid metabolism and regulation of the immune responses [73]. Interestingly, experimental data indicated that the administration of *B. thetaiotaomicron* reduces the de novo sphingolipid pathway, whereas ceramides increase in the liver of host mice, confirming the role of gut-derived bacterial sphingolipids on host lipid metabolism [74]. Bacteroides belonging to the gut microbiome favors symbiosis with the host; accordingly, a reduction in bacterial sphingolipids is associated with inflammatory bowel disease and increased host-produced sphingolipid amounts in the human intestine [75]. In addition, undegraded dietary sphingolipids in the distal small intestine may be metabolized by the gut microbiome into other bioactive lipids with biological activities [76].

Data from the Healthy Life in an Urban Setting study demonstrated that sphingolipids regulate the relationship between the gut microbiome and incident T2D, evidencing a dominance of ceramides over more complex sphingolipids [77]. Different lipids were associated with gut microbial species in children with T2D and metabolic syndrome; in particular, some ceramide species were positively associated with *Enterobacter hormaechei* [78]. A relationship among gut microbiota richness, ceramides, and T2D risk was also observed in overweight/obese adults, with ceramides being inversely correlated with gene richness [79]. Thus, these data strongly suggest the contribution of the gut microbiota to the dysregulation of lipid metabolism in metabolic disorders. In agreement with this evidence, an interventional study on healthy subjects evidenced that the intake of omega-3 and fiber supplementation reduces specific ceramide ratios of Cer16:0, Cer24:0, and Cer24:1 (ceramide predictors of CV mortality and major CV adverse events) in association with a decrease in the abundance in *Colinsella* and increases in *Bifidobacteriuim* and *Coprococcus 3* and short-chain fatty acid production [80]. These results reinforce the potential of exploiting dietary interventions to modulate the levels of these lipid biomarkers linked to CV risk, attributed, almost in part, to a change in the microbiome composition and its effects on the production of metabolites.

## 5. Discussion and Conclusions

Ceramides are emerging as key factors in the onset and development of obesity and T2D, also in view of their connection with inflammation and the contribution to the pathophysiology of obesity and IR. Moreover, ceramides are also at the crossroad between diet and obesity and T2D. There are nutrients and dietary patterns that negatively impact the ceramide profile (e.g., fructose, and the Western diet), whereas other foods are able to counteract adverse ceramide synthesis (e.g., ω-3 PUFAs, resveratrol, vitamin D, and the Mediterranean and the Nordic diets). However, although not a single cause, a number of mechanistic and functional implications have been identified to support the causal link between altered ceramide levels and obesity and T2D pathophysiology (including promotion of IR and apoptosis, increased inflammation and oxidative stress, and impairment of mitochondrial function), and whether ceramide nutritional modulation may represent an effective tool to improve metabolic health remains to be definitively established. In fact, these molecules can act either with favorable or adverse effects under different pathophysiological states (e.g., they can increase the production of apoptotic ceramides, with benefits in the cancer setting but not for pancreatic cells). Thus, the complexity of ceramide biosynthesis, the different interactions among the numerous ceramide species, and the intricate machinery of regulatory pathways still make the comprehension of ceramide homeostasis and its implications in the balance of metabolic health and disease challenging.

Moreover, current evidence, focusing on the application of nutritional strategies for modulation of the lipid asset to counteract the metabolic risk, still present limitations, implying caution and an evaluation of the weight of evidence in the conclusion derived by the interpretation of results, given the following aspects:

- Small sample sizes in several intervention trials;
- Short intervention durations (2–8 weeks);
- Lack of long-term clinical endpoints.

Nonetheless, the passage of the Cer measure in clinical laboratory practice might be feasible in terms of translational tools [2,17]. Surely, to achieve this goal, several technical and clinical aspects need to be improved; instrumentation is specialized, not largely diffused in all clinical laboratories, and requires accurate maintenance, technical expertise, and skilled operators, and standardization and analytical reproducibility must be further improved, costs still need to be elevated, clinical validation in different populations has not yet been concluded, and the interpretation of results remains complex for clinicians [2,17]. Nonetheless, one instrument consents to run hundreds of samples/day, which may be a way to reduce costs, as it has been done in the past for other tests (e.g., hormones, such as vitamin D) [2,17]. In this context, the identification of a reduced number of significant ceramides (and/or their ratios in panels/scores; e.g., the validation of CERT1 and/or CERT2 in different clinical settings) may favor the translational process, simplifying clinicians’ ability to better interpret the complexity of data obtained through this type of analysis.

CERT2, in particular, has been associated with mortality in patients with stable or acute coronary artery disease or heart failure and is related to different biomarkers of inflammation, myocardial necrosis, myocardial dysfunction, renal dysfunction, and dyslipidemia [81,82,83]. Moreover, CERT2 also improves cardiovascular risk prediction in primary prevention (*n* = 7324, followed up for 10 years for incident coronary heart disease, cardiovascular disease, major adverse cardiovascular event, stroke, and heart failure) [84]. CERT2, after a multivariate adjustment, predicted new-onset hypertension in the population from the 2002 FINRISK (Finnish non-communicable risk factor survey; 7722 participants, follow-up of 10 years) study [85]. In the same cohort, CERT scores (CERT1 and CERT2) were associated with prevalent rheumatoid arthritis and obstructive pulmonary disease, evidencing the importance of considering inflammatory risks, as well the possibility for implications of use, in smokers [86,87].

For these reasons, further studies must be performed in the future to assess the possibility to plan tailored dietary patterns able to modify the ceramide profile in order to better outline metabolic risk and improve therapeutic management and prognosis in nutrition-related metabolic conditions, with the aim of correctly translating ceramide evaluations from the bench to the bedside and effectively contributing to an improvement in the health of patients with obesity and T2D.

## Figures and Tables

**Table 1:** The three key pathways of sphingolipid metabolism.

De novo Synthesis Pathway	Salvage Pathway	Catabolic Pathway
*Serine + Palmitoyl-CoA*	*Sphingomyelin*, *Glycoshingolipids*	*Sphingomyelin*
**SPTLC 1–3 (sub-unit serine palmitoyl transferase)**	**SMase (Sphingomielinase)**	**SMPD 1–3 (sphingomyelin phosphodiesterase)**
*3-Ketosphinganine*	*Ceramide*	*Ceramide*
**KDSR (3-ketosphinganine reductase)**	**ASAH (Ceramidase)**	
*Sphinganine*	*Sphingosine*	
**CERS 1–6 (Ceramide synthase)**	**CERS 1–6 (Ceramide synthase)**	
*Dihydroceramide*	*Ceramide*	
**DEGS 1 (Dihydroceramide desaturase)**		
*Ceramide*		

*Enzymes in bold; precursor/product of the reaction in Italic.*

## Data Availability

No new data were created or analyzed in this study.

## Abbreviations

The following abbreviations are used in this manuscript:

MI	Myocardial infarction
BMI	Body mass index
IGT	Impaired glucose tolerance
IFG	Impaired fasting glucose
HOMA	Homeostatic Model Assessment for Insulin Resistance
CVD	Cardiovascular disease
Cer	Ceramide
CerS	Ceramide Synthases
COX-2	Cyclooxygenase-2
ER	Endoplasmatic Reticulum
IR	Insulin Resistance
MHO	Metabolically healthy obesity
MUO	Metabolically unhealthy obesity
NF-κB	Nuclear Factor κ B
PC	Phosphatidylcholine
PI3K	Phosphatidylinositol 3- Kinase
PKB	Protein kinase B
PKC	Protein kinase C
PUFA	Unsaturated fatty acids
RSV	Resveratrol
SDS	Strong Heart Study
SFA	Saturated fatty acids
SHFS	Strong Heart Family Study
SM	Sphingomyelin
SPT1	Serine palmitoyl transferase
T2D	Type 2 diabetes
TLR4	Toll-like receptor 4
VLDL	Very low-density lipoproteins
WAT	White adipose tissue
